# A Patient With Foot Pain Found to Have Leriche Syndrome: A Case Report and Brief Review of the Literature

**DOI:** 10.7759/cureus.39199

**Published:** 2023-05-18

**Authors:** Moshe Bengio, Quan Ta, Glenn Goodwin, Megan De Kok, Alexander J Scumpia

**Affiliations:** 1 Medical School, Nova Southeastern University Dr. Kiran C. Patel College of Osteopathic Medicine, Davie, USA; 2 Emergency Medical Services, Hatzalah South Florida Emergency Medical Services, Miami, USA; 3 Emergency Medicine, HCA Florida Aventura Hospital, Aventura, USA; 4 Surgery, Florida Atlantic University Charles E. Schmidt College of Medicine, Boca Raton, USA; 5 Osteopathic Medicine, Touro College of Osteopathic Medicine, New York, USA; 6 Medicine, Rocky Vista University College of Osteopathic Medicine, Englewood, USA; 7 Emergency Medicine, Lakeside Medical Center - Health Care District Palm Beach County, Belle Glade, USA

**Keywords:** catheter-directed thrombolysis, aortoiliac disease, infrarenal aortic thrombus, limb ischemia, kissing stents, peripheral artery disease, leriche syndrome

## Abstract

Leriche syndrome, a rare and critical complication of peripheral arterial disease (PAD), affects the distal abdominal aorta (infrarenal) and, similar to PAD, is a result of plaque buildup in the arterial lumen. The Leriche syndrome triad includes claudication in the proximal lower extremity, decreased or absent femoral pulses, and, in some cases, impotence. This article presents a patient with an atypical presentation of foot pain who was subsequently found to have Leriche syndrome. The patient was a 59-year-old female, a former smoker, who presented to the emergency department (ED) with atraumatic, acute right foot pain. All right lower extremity pulses were faintly audible on bedside Doppler. Computed tomography with angiography of the abdominal aorta revealed a Leriche-type occlusion of the infrarenal abdominal aorta and left common iliac and a 10 cm right popliteal arterial occlusion. Pharmacological anticoagulation was initiated by the ED. Definitive treatment in this patient included catheter-directed tissue plasminogen activator lysis to the thrombus on the right and placement of kissing stents in the distal aorta without complication. The patient made an excellent recovery and had a complete resolution of her symptoms.

PAD is an omnipresent condition and, when untreated, can result in a myriad of high mortality and morbidity conditions such as Leriche syndrome. Collateral vessel formation can make the symptoms of Leriche syndrome vague and inconsistent, often making early recognition difficult. Optimal outcomes hinge on the clinician’s ability to efficiently recognize, diagnose, stabilize, and coordinate multidisciplinary involvement of vascular and interventional radiology specialties. Case reports such as this one help to illuminate some of the more infrequent presentations of Leriche syndrome.

## Introduction

Leriche syndrome, also known as aortoiliac occlusive disease, is a relatively rare and critical form of peripheral artery disease (PAD) [1]. Studies estimate that between 5% and 10% of individuals in the United States (US) over the age of 40 have some form of PAD, with 1.3% of the US population suffering from a severe stage of critical limb ischemia (CLI) [1]. Untreated PAD can lead to CLI, placing these individuals at a greater risk for major amputation (100 per 1,000 person-years) and all-cause mortality (183 per 1,000 person-years) when compared to the less severe forms of PAD (26 and 81 per 1,000 person-years, respectively) [2]. Positive outcomes related to Leriche syndrome hinge on prompt stabilization measures and definitive surgical intervention. This report presents an atypical presentation of Leriche syndrome from an emergency department (ED) perspective and then reviews the literature for key points on pathophysiology, history and physical examination findings, diagnostic modalities, and cutting-edge interventions.

## Case presentation

**Figure 1:**
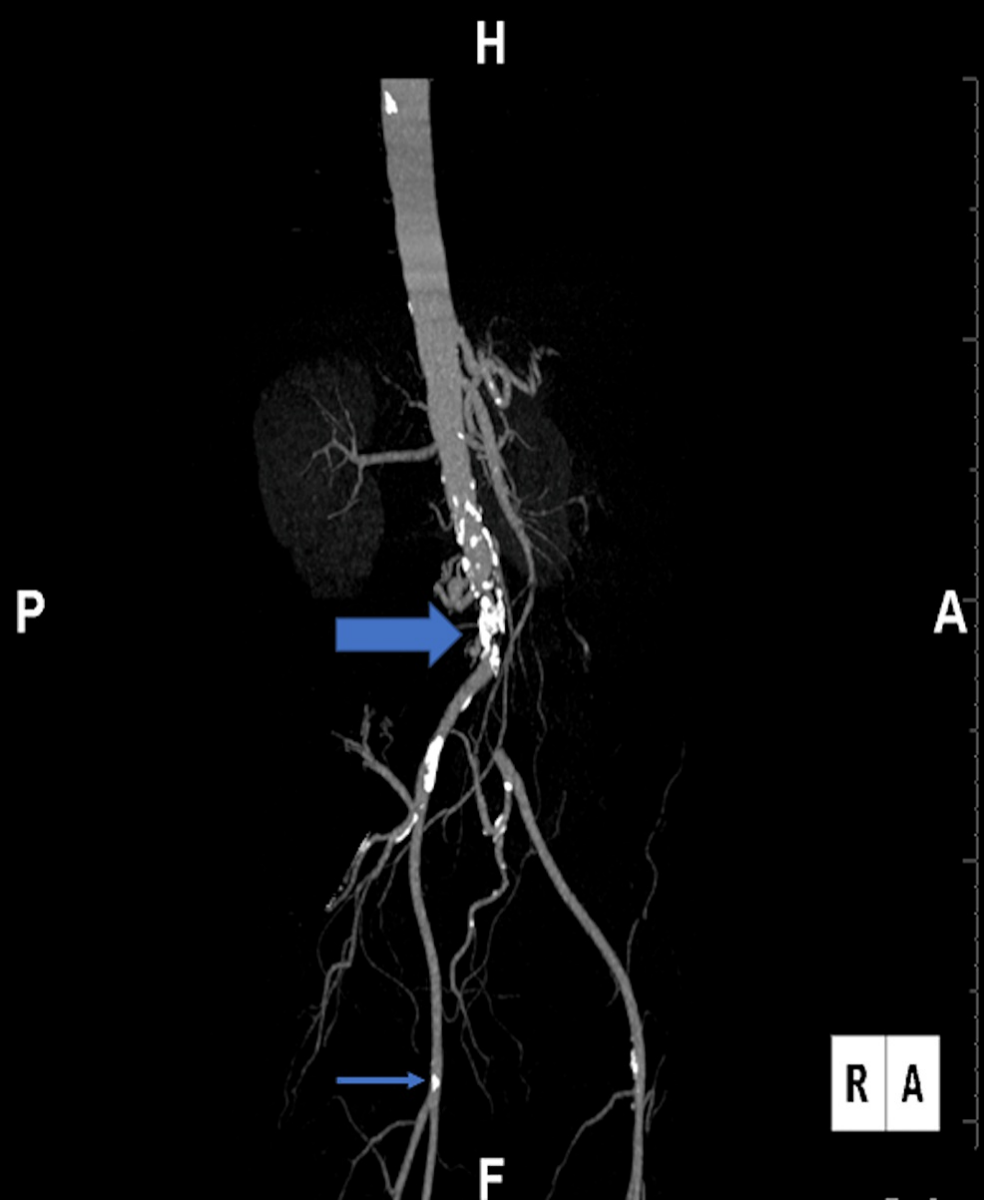
Three-dimensional computed tomography of the abdomen with aortic angiography and lower extremity runoff. Leriche-type occlusion of the distal abdominal aorta and the left common iliac artery (thick blue arrow). A 10 cm occlusion of the right popliteal artery (thin blue arrow) and below-knee trifurcation, with reconstitution of the proximal anterior tibial and mid-posterior tibial arteries.

A 59-year-old female presented to our ED with a one-day history of atraumatic, acute right foot pain. Vital signs were grossly unremarkable. The patient denied weakness but stated occasional right foot numbness. Physical examination showed no loss of mobility, discoloration, or temperature change in the right lower extremity (RLE); however, femoral, popliteal, pedal, and posterior tibial pulses could not be palpated as they were faintly audible on bedside Doppler. The patient was a former heavy smoker and stated she had quit two years ago. ED laboratory results as well as a three-view right foot X-ray were unremarkable. Arterial duplex ultrasonography of the patient’s RLE demonstrated diffuse monophasic waveform and diminished arterial flow velocities suggestive of possible iliac stenosis versus occlusion. Computed tomography angiography (CTA) of the abdominal aorta with bilateral lower extremity runoffs was performed. The CTA, as seen in Figure 1, revealed complete occlusion of the infrarenal abdominal aorta and the left common iliac artery. Interestingly, the right common iliac was patent. Lastly, a 10 cm right popliteal arterial occlusion at the level of the distal femoral artery was also discovered. The right popliteal occlusion was the most likely reason for the acute right foot pain.

**Figure 2:**
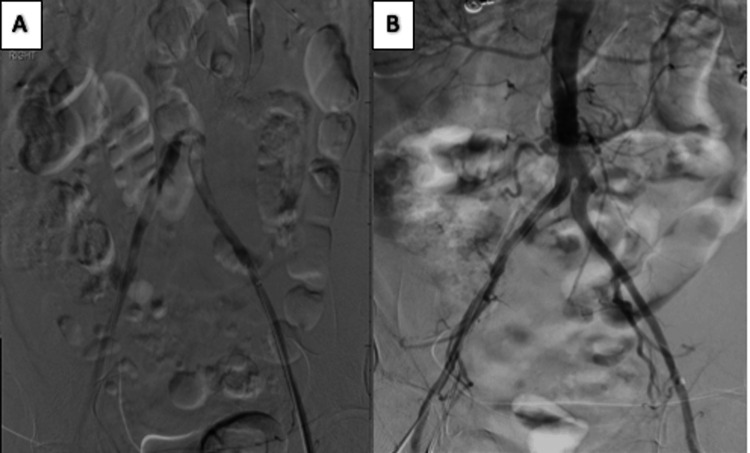
Digital subtracting angiography (DSA). A: DSA before stent placement with absent flow in the distal aorta. B: DSA post-tPA and stent placement demonstrating a patent distal aorta.

Vascular surgery and interventional radiology (IR) were emergently consulted, and the patient was started on enoxaparin (1 mg/kg) in the ED. The patient was deemed a good candidate for IR intervention. Catheter angiography was performed, which again demonstrated the distal aortic occlusion, left common iliac thrombus, and right popliteal thrombus. Catheter-directed tissue plasminogen activator (tPA) lysis was initiated with attention to the right popliteal thrombus at 1 mg/hour for 15 hours. Additional tPA lysis was performed to treat the left iliac thrombus. Subsequently, the aortic occlusion was treated with bilateral aortoiliac kissing stents showing patent bilateral common, internal, and external iliac arteries. Figure 2 demonstrates occlusion resolution pre and post-procedure. Right popliteal artery thrombus also resolved with patency and brisk perfusion of the RLE, demonstrating retrograde perfusion of the distal posterior tibial artery. The patient made an excellent recovery and is doing well according to a telephonic follow-up several weeks later.

## Discussion

**Figure 3:**
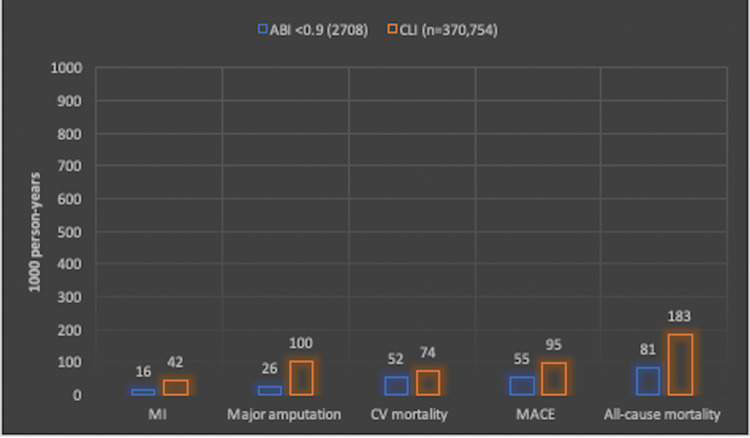
PAD morbidity and mortality per 1,000 person-years. The minimal definition for PAD is ABI <0.9. The CLI group, the severest form of PAD, had a significantly higher relative risk (with 95% confidence intervals) for MI 2.63 (1.49, 4.64), amputation 3.85 (2.52, 5.87), CV mortality 1.42 (1.01, 2.01), MACE 1.73 (1.25, 2.38), and all-cause mortality 2.26 (1.77, 2.89) when compared to the PAD ABI <0.9 group using data reported per 1,000 person-years [2]. PAD = peripheral arterial disease; ABI = ankle-brachial index; CLI = critical limb ischemia; MI = myocardial infarction; CV = cardiovascular; MACE = major adverse cardiac event

Rene Leriche, of vascular specialty fame, was the first to characterize Leriche syndrome after observing patients from 1923 to 1940 with an atypical form of PAD. In 1948, Leriche and Morel documented a variety of patients with a common triad of claudication, absent femoral pulses, and erectile dysfunction. These patients were in the 30-40-year age range and were nearly all males. They were subsequently discovered to have an aortic infrarenal thrombotic obstruction, and the diagnosis of Leriche syndrome was born [3]. Similar to other forms of PAD, this syndrome occurs in the setting of severe atherosclerosis. The prevalence of Leriche syndrome is unknown given that many cases are asymptomatic, but PAD, the etiological agent of Leriche syndrome, has a prevalence of approximately 115 million worldwide with 70,000 deaths in 2019 [1]. The data is skewed toward the older age range, and some studies suggest that incidence doubles each decade over the age of 40 [1]. The ankle-brachial index (ABI) of <0.9 is well-substantiated in satisfying the minimal criteria for PAD [2]. CLI is a severe form of PAD that results in an array of clinical manifestations, including claudication, sensory issues, and skin ulcerations [1,2]. In the United States alone, studies estimate the prevalence of PAD to range between 5% and 10%, with 1.3% suffering from CLI [1]. In a systematic review published in 2020, from a data pool ranging from 2003 to 2017, patients with CLI had a significantly higher relative risk for myocardial infarction, major amputation, cardiovascular mortality, major adverse cardiac event, and all-cause mortality when compared to patients without CLI but an ABI of <0.9 (Figure 3) [2].

Patients with any form of PAD share similar risk factors, including a history of increased age over 70, diabetes mellitus, dyslipidemia, hypertension, tobacco use, hyperglycemia, homocysteine metabolic derangements, and family history [4]. In typical PAD, the most implicated caudal vessels include tibial, peroneal, superficial femoral, popliteal, common femoral, and aortoiliac arterial junctions [5]. Once sclerosed, the vessels often result in ipsilateral claudication symptoms at the level of the foot, calves, thigh, buttock, and hip, respectively [5]. If plaque buildup develops at or near the infrarenal aortic bifurcation, it can lead to the rare condition of Leriche syndrome, which occurs once those vessels are occluded [5]. Most of these patients are elderly with a history of advanced atherosclerotic disease, and female in acute cases. More recent data suggest that the triad of claudication, absent femoral pulses, and erectile dysfunction is more commonly seen in patients with chronic onset of atherosclerosis [4].

Leriche syndrome can be divided into three classifications based on the extent of the spread of the occlusion (Table 1) [5]. Proximal occlusion typically does not result in downstream ischemia because in many cases, collateral vessels will form to supply distal circulation [6]. Commonly, collateral formation follows the superior mesenteric artery into the rectal artery supply, to the internal iliac artery or obturator artery, and to the common femoral artery [6]. Other more proximal reconstitution routes are the costal arteries to superior gluteal arteries. A rare but nonetheless alternative collateral formation is the Winslow pathway, which follows the subclavian artery into the thoracic, epigastric, and then external iliac [7].

**Table 1:** Classification of Leriche syndrome based on occlusion extension. Leriche syndrome has three classes based on the extent of the occlusion with minimum criteria involving the infrarenal aorta and common iliac artery (class I). Further extension into the external iliac artery is class II. In class III, the occlusion reaches the femoropopliteal artery [5].

Occlusion location/Extent		Class
Infrarenal aorta	Common iliac artery	I
	External iliac artery	II
	Femoropopliteal artery	III

When reconstitution of arterial flow occurs, Leriche syndrome can be asymptomatic [8]. Unfortunately, undiagnosed and untreated Leriche syndrome can lead to catastrophic consequences and mortality [9]. In symptomatic cases, patients may have pain, paresthesia, paralysis, pulselessness, pallor, weakness, or impotence. The location of the symptoms or pulselessness depends on the extent of the vessels affected and collaterals formed, but will typically include hip and buttocks pain accompanied by absent or diminished femoral pulse [4].

Diagnosis of Leriche syndrome should be confirmed once aortic dissection has been ruled out [4]. Leriche syndrome is also commonly mistaken for lumbar nerve pathologies such as cauda equina or conus medullaris syndrome, given the symptomatology and patent pedal pulses [3].

Diagnosis of suspected lower limb PAD and Leriche syndrome should begin with the ABI, with scores <0.9 prompting an arterial ultrasound Doppler study of the lower extremity. The Doppler is an inexpensive, easy, and fast test with no radiation risk to accurately assess vascular flow, especially in the presence of circumferential calcifications. However, with collateral flow, the Doppler results could be falsely reassuring. Therefore, CTA of the lower extremities commonly follows suit for precise surgical planning and management [1,4,10].

Once confirmed, vascular surgery should be consulted for definitive management. In the interim, in the presence of acute symptoms with impending limb ischemia, ED treatment involves an initial heparin bolus of 80 U/kg, followed by a continuous infusion of 18 U/kg/hour [11].

Standard surgical treatments include aortobifemoral bypass (more common) and axillofemoral bypass with or without endarterectomy [12,13]. Aortobifemoral bypass patency rates were reported to be up to 85% and 75%, for five and 10 years, respectively [14,15]. In those with significant comorbidities, percutaneous transluminal angioplasty with or without stenting is another option [4]. One of the more cutting-edge modalities is the covered endovascular reconstruction of aortic bifurcation (CERAB) technique. The original stent procedure is the double kissing stent, in which both proximal portions of the stents before the bifurcation overlap, and the distal halves descend down a respective iliac vessel by the internal stent piercing the external stent just before the bifurcation [16]. In CERAB, there are three covered stents, with the main stent in the distal aorta only. The two other stents are placed within the aortic stent and their respective iliac arteries. By closely mimicking the natural anatomy, CERAB is theorized to cause less turbulence or niduses for thrombosis [16]. Initial studies are demonstrating the potential for better outcomes with CERAB [16].

## Conclusions

This case illustrates the importance of proper history taking and physical examination in patients presenting with atraumatic pain. Foot pain is a ubiquitous complaint in the ED which often does not command invasive testing or management, often leading to delayed diagnoses of not only Leriche syndrome but PAD in general. While Leriche syndrome is a relatively rare condition, PAD is an extremely common condition for a typical ED patient, necessitating the ED physician to be aware of the myriad of complications. Given the high morbidity and mortality rates, positive outcomes hinge on swift diagnosis and initial stabilization techniques. Unfortunately, Leriche syndrome can also be masked by collateral vessel formation circumventing the occlusion, resulting in asymptomatic progression of the disease for years before presentation. It becomes imperative for the emergency physician to efficiently recognize, diagnose, image, stabilize, and coordinate vascular and interventional specialties consultation to recover the affected extremity or prevent progression.
